# Optimized clustering method for spectral reflectance recovery

**DOI:** 10.3389/fpsyg.2022.1051286

**Published:** 2022-11-23

**Authors:** Yifan Xiong, Guangyuan Wu, Xiaozhou Li, Xin Wang

**Affiliations:** ^1^Faculty of Light Industry, Qilu University of Technology, Shandong Academy of Sciences, Jinan, China; ^2^State Key Laboratory of Bio-based Material and Green Papermaking, Qilu University of Technology, Shandong Academy of Sciences, Jinan, China

**Keywords:** spectral recovery, dynamic partitional clustering, color space, camera responses, spectral reflectance

## Abstract

An optimized method based on dynamic partitional clustering was proposed for the recovery of spectral reflectance from camera response values. The proposed method produced dynamic clustering subspaces using a combination of dynamic and static clustering, which determined each testing sample as *a priori* clustering center to obtain the clustering subspace by competition. The Euclidean distance weighted and polynomial expansion models in the clustering subspace were adaptively applied to improve the accuracy of spectral recovery. The experimental results demonstrated that the proposed method outperformed existing methods in spectral and colorimetric accuracy and presented the effectiveness and robustness of spectral recovery accuracy under different color spaces.

## Introduction

Spectral reflectance describes wavelength-dependent reflectance functions and determines the intrinsic color characteristics of object materials (Liang and Wan, 2017). Spectral matching can successfully predict the color appearance of a scene under arbitrary environmental conditions, which has led to its widespread use in high-fidelity reproduction (Liu et al., 2019), remote sensing (Lin and Finlayson, 2020), cultural heritage (Liang, 2011), medical diagnosis (Depeursinge et al., 2009; Nishidate et al., 2013; Xiao et al., 2016), etc. (Wu et al., 2016; Jinxing et al., 2022). The spectral acquisition can be directly measured by spectrophotometers or multispectral cameras; however, it is often accompanied by the limitations of their complexity and inconvenience with less practical application (Liu et al., 2017; Wu, 2019; Molada-Tebar et al., 2020). Alternatively, camera response values can be easily acquired using digital cameras or scanners. Hence, it is valuable to recover spectral reflectance from camera response values in this direction (Wang et al., 2020).

The camera response values are three integral values representing the object color information under a specific environmental condition; therefore, spectral recovery from camera response values is generally used to solve an ill-posed inverse problem. Several methods, such as the pseudo-inverse (PI) method (Babaei et al., 2011; Wu et al., 2015; Maali Amiri and Fairchild, 2018), compressive sensing (CS) (Zhang et al., 2014; Dafu et al., 2017), Wiener estimation (Stigell et al., 2007; Nishidate et al., 2013), principal component analysis (Liang, 2011), and convolutional neural networks (Xiong et al., 2022), are being applied directly to recover the spectral reflectance. Accurate spectral recovery methods are a more meaningful and valuable solution for practical applications; therefore, the proposed strategies have been mostly concerned with optimal sample selection and processing. The greater the similarity between the training and testing samples, the better the accuracy of the spectral recovery. Li et al. (2013) proposed a spectral recovery method based on a locally linear approximation by weighting the nearest neighbors. Cao et al. (2017) attempted to recover the spectral reflectance from camera response values using local weighting modes based on color differences. Zhang et al. (2016) selected training samples by drawing a sphere in the camera response value space and used Wiener estimation to recover the spectral reflectance. Wang et al. (2020) applied the sequential weighted nonlinear regression method to recover the spectral reflectance from the camera response values, considering colorimetric and spectral errors. Wu et al. (2021) presented a compressive sensing-based spectral sparse recovery method that uses sparse basis functions. These methods could reasonably produce spectral recovery accuracy using predefined parameters; however, they are insufficient for selecting the training samples for each testing sample characteristic. Kwon et al. (2007) utilized Lloyd’s algorithm based on K-means (KM) clustering to iteratively divide the entire training sample into subgroups with similar colors and recover the spectral reflectance using the principal components obtained by subgroups. Xu et al. (2017) employed the KM clustering algorithm to partition multispectral images and extract training samples from an art painting, which selected the representative sample in each cluster for spectral reflectance recovery. Although the above methods achieve adaptive sample selection strategies using the clustering method, the characteristics of each testing sample were not considered in the clustering process. Thus, it is necessary to develop an optimized clustering method to adaptively select the training samples based on the characteristics of each testing sample.

This paper has presented an optimized method based on dynamic partitional clustering for spectral reflectance recovery from camera response values. The novelty of the proposed method is that it produces dynamic clustering subspaces by utilizing a combination of dynamic and static clustering, which determines each testing sample as *a priori* clustering center to the sample characteristics of the clustering subspaces through competition. After acquiring the training samples, the spectral reflectance is recovered using the Euclidean distance weighted and polynomial expansion models in the clustering subspace. The effectiveness of the proposed method is compared with existing methods in terms of spectral and colorimetric accuracy, and the effectiveness and robustness of the proposed method for spectral recovery accuracy under different color spaces are presented.

## Materials and methods

The incoming light was reflected by the object’s surface through the camera, which converted it to an electrical signal and quantified the camera response values based on the human perception of colors. The camera response values were calculated by the combination of the camera sensitivity functions *q*(λ), illuminant spectral power distribution **I**(λ), and object surface reflectance *r*(λ), which could be described using the integral process.


(1) $$Y=\int_{\lambda_{min}}^{\lambda_{max}}I\left(\lambda \right)r\left(\lambda \right)q\left(\lambda \right)d\lambda +\varepsilon$$

where ***Y* = [*r, g, b*]*^T^*** is the corresponding camera response value, the superscript “T” is the matrix transposition, λ is the human visible wavelength that includes the range from λ _*min*_ to λ _*max*_, and ε is the camera system noise. The camera system noise was negligible owing to measurement difficulty (Arad and Ben-Shahar, 2017); thus, Equation (1) can be expressed simply in matrix notation:


(2) Y=MR

where ***M*** denotes the integration matrix that calculates the camera sensitivity function *q*(λ) and the illuminant spectral power distribution **I**(λ) and ***R*** represents the spectral reflectance. The reverse solution was to recover the spectral reflectance from the camera response values formulated by Equation (3)


(3) R=QY

where ***Q*** is the transformation matrix. The main goal of this study was to minimize the spectral recovery error from the camera response values.


(4) $$R=arg\underset{R}{min}{∥\;R-QY\;∥}_{l_{1}}$$

where ∥⋅∥*l*_1_ denotes the *l*_1_ Frobenius norm of the matrix. The spectral recovery process was implemented by treating all training samples equally, which could theoretically be optimum for all testing samples other than each sample. This suggested that the transformation matrix *Q* could be obtained by considering each testing sample characteristic, and the result of spectral recovery would be optimal. Thus, the problem of spectral reflectance recovery was transformed into the optimization of the transformation matrix *Q*. Therefore, the optimal sample selection and processing were adaptive to each testing sample characteristic, making a reasonable choice for producing the optimal transformation matrix.

## The proposed method

**FIGURE 1:**
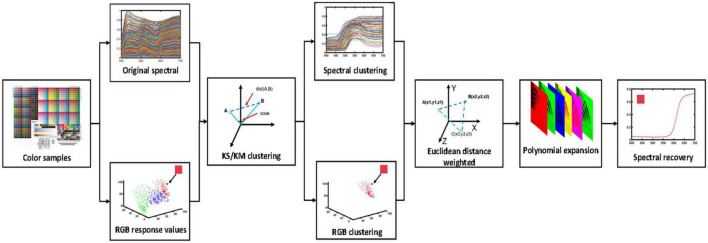
Schematic illustration of the optimized clustering method for spectral reflectance recovery.

In this section, an optimized clustering method for spectral reflectance recovery was proposed for camera response values, which is schematically illustrated in Figure 1. The widely used pseudo-inverse (PI) method is a straightforward method to present the relationship between the camera response values and the sample reflectance, which was used to verify the sample selection and processing for the method proposed in this study.

### Acquiring camera response values

The camera response values of the simulation experiments were calculated instead of those extracted from the images because the spectral power distribution of the light source and the spectral sensitivities of the camera could be measured (Arad and Ben-Shahar., 2017). Finally, the response value of the simulated camera was integrated by the measured spectral sensitivities of the camera, spectral power distribution of the light source, and spectral reflectance. For the real experiment, the raw images (“CR2” format file) were acquired using a Canon EOS 5D Camera, controlled by software (Digital Photo Professional 4). The Tagged Image File Format (TIFF) can be easily converted from the raw images, which is used to acquire the corresponding red, green, and blue (RGB) response values.

### Selecting the optimal local training samples

For a given training sample (**x_1_…** **x_m_**) in RGB color space, each *x*_*i*_ ∈ **Y**_**T***rain*_, where m is the number of training samples. Let (**p_1_…** **p_n_**) ∈ **Y**_**T***est*_ be the testing samples in RGB color space, with **p_e_** ∈ **Y**_**T***est*_, where n is the number of testing samples. The set of clustering centers can be expressed as (**u_1_u_2_… u_l_**) ∈ **L**, where l indicates the number of clustering centers; **u_1_** is a fixed point, which is the testing sample considering the chromaticity characteristics as the prior clustering center, in this work, **u_1_** = **p_e_**. The other clustering centers are the dynamic random points based on the training samples, which are obtained by the KM clustering algorithm. The partition information of subspaces *C* is determined by the division of the clustering centers. Minimizing a cost function *W* (*C, L*) shows the natural structure of **Y**_**T***rain*_.


(5) $$W\left(C,L\right)=min_{C}\sum_{L=1}^{a}\sum_{x_{i}\in C_{a}}∥x_{i}-u_{a}∥(i=1,2,\ldots m,\;a=1,2,..l)$$

where the subscript ***i*** denotes the ***i*th** sample of training samples; the subscript ***a*** denotes the ***a*th** sample of the clustering centers; the *C_a_* denotes the ***a*th** division subspace; and the ∥.∥ represents a different way of partition, which is cosine angle or Euclidean distance. Two approaches were used as criteria to determine the distance. The cosine angle distance was used as the basis for partitioning, which was the K-mean angle similar (KS) clustering strategy selected in this study. The ∥ *x*_*i*_ − *u*_*a*_ ∥ can be demonstrated in Equation (6).


(6) $$∥x_{i}-u_{a}∥=acos\left(\frac{<x_{i},u_{a}>}{∥x_{i}∥∥u_{a}∥}\right)$$

The Euclidean distance was used as the basis for partitioning, which was the KM clustering strategy selected in this study. The ∥ *x*_*i*_ − *u*_*a*_ ∥ can be demonstrated in Equation (7).


(7) $$∥x_{i}-u_{a}∥=\sqrt{\sum_{t=1}^{m}{\left(x_{i}-u_{a}\right)}^{2}}$$

The new partition was generated once the way of classification was determined.


(8) $$J_{t}=\frac{1}{h_{a}}\sum C_{a}\left(a=2,3,\ldots .l\right)$$

where *J_t_* represents the clustering centers of the subspaces ***C***, which together with the testing samples **p_e_** forms the set of clustering center ***L*** and **h**_*a*_ represents the number of samples in each division subspace *C_a_*. In this process, the subspace where the test sample was located did not seek the average value, whereas the other subspaces regenerated the center point to obtain the optimal local subspace. Let the cosine angle or Euclidean distance be a distance function. It can easily calculate the distance between the clustering centers and training samples. Equations (6) and (7) were substituted into Equation (5) to obtain the new subspace information. After processing, the subspaces other than the subspace where the test sample (the first clustering center) was located were averaged to obtain the remaining new clustering centers. Finally, the new clustering centers L were obtained and substituted into Equation (5) to obtain the final subspace.


(9) $$\Omega =\left[x_{1},x_{2},x_{3}\ldots x_{N}\right]\left(N<m\right)$$

where ***N*** is the number of optimal local training samples and Ω is the subspace where the test sample as the clustering center is located. The optimal local training samples were located in the subspace Ω from the training samples according to the testing sample. This was the methodological procedure used in this study.

### Calculating weighting matrix

After the optimal local training samples were acquired, the Euclidean distance of RGB was used for the weighting function because the similarity of the target samples also played an important role (Maali Amiri and Fairchild, 2018; Jinxing Liang et al., 2019).


(10) sj=(rtest-rtrain,j)2+(gtest-gtrain,j)2+btest-btrain,j2 (j=1,2,…,N)

where **r**_**t***est*_, **g**_**t***est*_, and **b**_*test*_ are the responses of the testing sample; *r*_*train,j*_, *g*_*train,j*_, and *btrain*,*j* are the responses of the optimal local training samples; ***N*** is the number of optimal local training samples; and the subscript ***j*** is the ***j*th** sample of Ω.


(11) $${\text{w}}_{j}=\frac{1}{s_{j}+\theta }\left(j=1,2,\ldots ,N\right)$$

where *w*_*j*_ represents the inverse of the Euclidean distance between the testing sample and the *j*th training sample; *θ* is a very small value in Equation (11), and *θ* = 0.001, which makes sure the denominator is not zero. It can also be expressed as a diagonal matrix *W*.


(12) $$W={\left[\begin{array}{cccc} w_{1} & 0 & \cdots & 0\\ 0 & w_{2} & 0 & 0\\ \vdots & 0 & \ddots & \vdots \\ 0 & 0 & \cdots & w_{N} \end{array}\right]}_{N\times N}$$

### Responses expansion

Response expansion is a common nonlinear technique for increasing the accuracy of recovery. It increases with the number of items until a specific value is reached. In this paper, items of polynomials were discussed. The 3-dimensional data of ***Y*** were mapped to higher dimensional data **Y**_**e***xp*_.


(13) $$Y_{exp}=[1,r,g,b,rg,rb,gb,r^{2},g^{2},b^{2},rg^{2},rb^{2},r^{2}g,r^{2}b,gb^{2},g^{2}b,$$

$$\;r^{3},g^{3},b^{3},rg^{3},r^{2}g^{2},rg^{3}rg^{3},r^{2}g^{2},rg^{3},rb^{3},r^{2}b^{2},r^{3}b,$$

$$\;r^{2}b^{2},r^{3}b,gb^{3},g^{2}b^{2},r^{3}b,r^{2}gb,rg^{2}b,rgb^{2},r^{4},g^{4},b^{4}]{}^{T}$$

where **Y**_**e***xp*_ is the expansion of RGB response values and the superscript “***T***” indicates the transpose of the matrix.

### Spectral recovery based on polynomial extension

Matrix ***Q*** was the unique transformation matrix of each testing sample after processing, and each testing sample had its optimal subspace; hence, each transformation matrix ***Q*** was calculated from the corresponding subspace.


(14) $$Q=R_{Train}W{\left(Y_{Train,exp}W\right)}^{-1}$$

where the superscript “–1” indicates the pseudo-inverse matrix operator, *R*_*Train*_ represents the selected optimal local training sample, and *Y*_*Train,exp*_ is the response value of the training samples after the polynomial expansion.


(15) $$R_{test}=QY_{Test,exp}$$

where *R_test_* denotes the reconstructed spectral reflectance of the testing sample and *Y*_*Test,exp*_ denotes the expanded response value vector of the testing sample.

## Experiment

The performance of our method was demonstrated by validating the experimental data, which was implemented in the simulated and real experiments. The color differences of the International Commission on Illumination (CIE DE76) and CIE DE2000 under the CIE 1964 standard observer functions and CIE A illuminant were calculated, which was used as the colorimetric metric. The root-mean-square error (RMSE) and goodness of fit coefficient (GFC) represented the deviation between the original spectral and recovered spectral, which were used as spectral metrics.


(16) $$RMSE=\sqrt{\frac{1}{m}{\left(R_{test}-R\right)}^{T}(\left(R_{test}-R\right)}$$

(17) GFC=RtestTR∥RtestTRtest∥.∥RR∥

where *R*_*test*_ represents the reconstructed spectral reflectance, *R* represents the original spectral reflectance, and *m* = 31; the superscript “**T**″ indicates transpose. The American National Standards Institute (ANSI) IT8.7/3 color chart (928 samples) was selected as the experimental dataset for the simulation and real experiments, which prints on the coated paper using the AccrioPress EasPrint C6100. The spectral information of the ANSI IT8.7/3 color chart was then acquired using the spectrophotometer X-rite eXact at 10 nm intervals in the 400–700 nm range. The 464 odd sets of IT8.7/3 data were selected as the training sample and the 464 even sets of the IT8.7/3 data were chosen as the test sample.

### The simulated experiment

**FIGURE 2:**
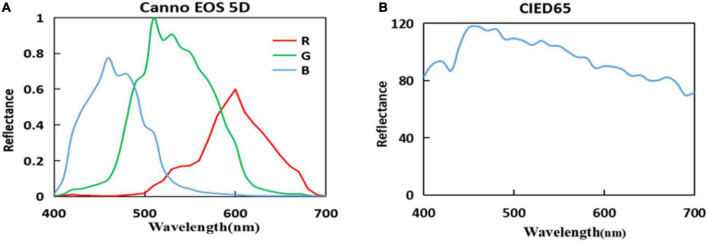
**(A)** Spectral sensitivities of the camera and **(B)** spectral power distribution of the International Commission on Illumination (CIE) Standard Illuminant D65.

To validate the performance of the methods, the simulated experiment was implemented by the simulated camera first. Owing to measuring the camera system noise difficulty, the calculation processing was not considered in the camera system noise in the simulated experiments. Figure 2A shows the spectral sensitivity of the Canon EOS 5D Camera (Jiang et al., 2013), and Figure 2B shows the spectral power distribution of the CIE Standard Illuminant D65.

**FIGURE 3:**
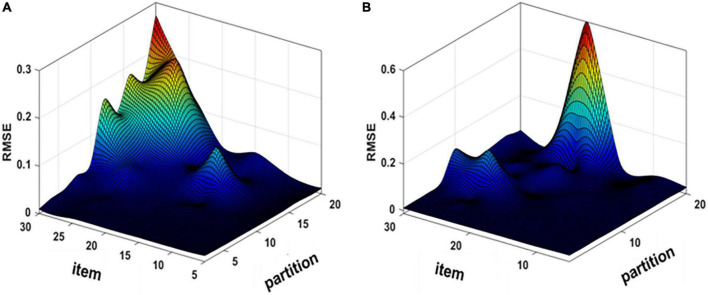
Mean root-mean-square error (RMSE) map of simulation experiment **(A)** K-mean angle similar (KS) and **(B)** K-means (KM).

To determine the optimal parameters of the proposed method, the different number of response expansion items and sample partitions were used directly to calculate the accuracy of spectral recovery. The response expansion items were selected from 5 to 30 with intervals of 5, and the partitions were tested from 2 to 20 with intervals of 2. Figures 3A,B show the RMSE under different response extension items and partitions by using the KS/KM strategy.

As can be seen from Figures 3A,B, the accuracy of spectral recovery is influenced by the number of response expansion items and sample partitions. The simulation results showed that the spectral recovery results were similar trends by using the KS/KM strategy. Therefore, the optimal response expansion items were 30 and the number of partitions was 2 for both KS and KM according to the experimental results.

As can be seen from Table 1, this study compares the accuracy of spectral recovery of the proposed method with seven other existing methods. The experimental results showed that our method has the lowest color difference, which means the proposed method has a fine colorimetric metric. The method used in this study is superior to other existing methods in RMSE and GFC, which also means the proposed method has fine spectral metrics. To visualize the recovery data and make the results more intuitive, this study used boxplots to analyze the spectral recovery accuracy.

**TABLE 1:** Simulated spectral recovery results of the proposed and other existing methods.

	**CIE DE76**								
**Results**	**Li**	**Wang**	**Wu**	**Zhang**	**Cao**	**Kwon**	**Xu**	**KS**	**KM**
Mean	0.57	1.30	0.74	0.60	14.13	0.52	0.31	0.10	0.10
Maximum	2.13	5.11	2.34	2.78	49.54	19.48	1.53	1.06	1.32
Minimum	0.07	0.05	0.01	0.01	0.22	0.01	0.01	0.00	0.00

	**CIEDE2000**								
	**Li**	**Wang**	**Wu**	**Zhang**	**Cao**	**Kwon**	**Xu**	**KS**	**KM**

Mean	0.43	0.99	0.63	0.40	10.42	0.35	0.24	0.07	0.07
Maximum	1.88	2.39	2.41	1.49	38.51	10.21	1.25	0.40	0.75
Minimum	0.06	0.04	0.00	0.01	0.25	0.01	0.01	0.00	0.00

	**RMSE (%)**								
	**Li**	**Wang**	**Wu**	**Zhang**	**Cao**	**Kwon**	**Xu**	**KS**	**KM**

Mean	0.71	0.69	0.93	0.83	5.49	0.55	0.46	0.15	0.16
Maximum	2.95	2.55	3.48	4.00	24.13	9.55	2.95	2.77	3.61
Minimum	0.06	0.007	0.10	0.14	0.18	0.004	0.004	0.01	0.01

	**GFC (%)**								
	**Li**	**Wang**	**Wu**	**Zhang**	**Cao**	**Kwon**	**Xu**	**KS**	**KM**

Mean	99.89	99.94	99.83	99.79	97.53	99.92	99.95	99.99	99.99
Maximum	100.00	100.00	100.00	100.00	100.00	100.00	100.00	100.00	100.00
Minimum	97.82	99.28	98.93	96.00	74.99	98.05	98.75	99.75	99.59

**FIGURE 4:**
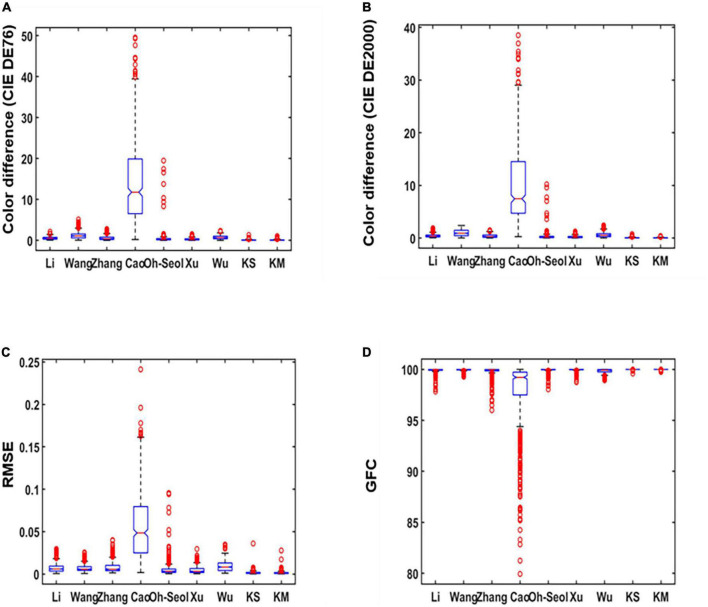
Boxplots of simulated experiment results: **(A)** The International Commission on Illumination (CIE) DE1976 color difference, **(B)** CIE DE2000 color difference, **(C)** root-mean-square error (RMSE), and **(D)** goodness of fit coefficient (GFC).

The results in Table 1 are used as original data to produce the boxplot. The boxplot is a standardized way of displaying the spectral recovery results, which are the minimum, maximum, median, and first and third quartiles. The value closest to the box indicates the best spectral recovery results while the value farther from the box indicates the worst spectral recovery results. The box of the boxplot of the proposed method is smaller than other methods and shows the best results in the maximum and mean. This signifies that our method recovers more samples with minor errors. As shown in Figures 4A,B, it can be observed that our method has the highest accuracy for both the average and minimum color difference values. Figures 4C,D show the smaller box, which means that the proposed method outperformed other existing methods in terms of spectral accuracy.

**FIGURE 5:**
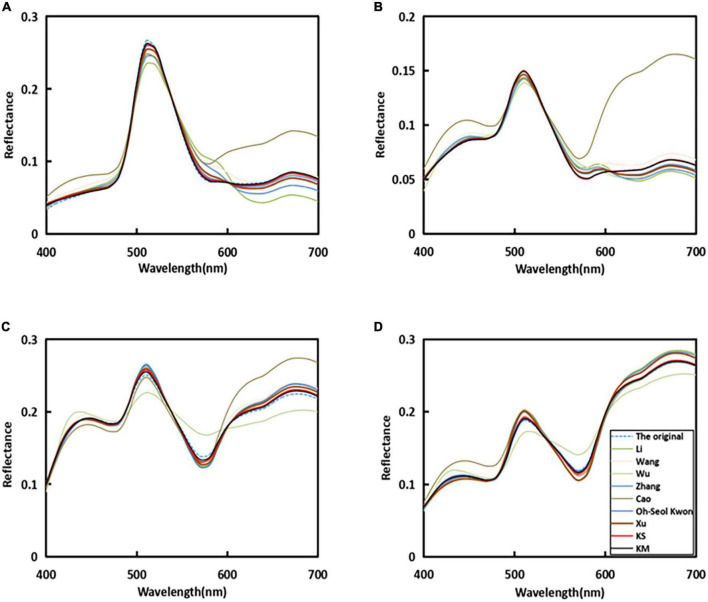
Reflectance recovery results from the proposed and existing methods with four randomly selected samples in the simulated experiment.

In Figure 5, it can be seen that the original sample spectral reflectance curve is set as a blue dashed line, and the line of our proposed method is set as distinct red and black colors. The recovered spectral curves of four randomly selected samples by using the proposed method and other existing methods were compared with the original spectral curves. It can be seen that the proposed method in this study is closer to the original sample curve among the four randomly selected plots.

### The real experiment

The real experiments were conducted in a dark room without interference from external light sources. To ensure accurate response values, a diffuse illumination environment was provided and the final data were obtained by software (Digital Photo Professional 4). The IT8.7/3 data were captured with a Canon EOS 5D; the camera’s ISO size is 50, the f-number aperture is F5.6, and the exposure time is 1/10 s. The real response values were extracted in sRGB color space.

**FIGURE 6:**
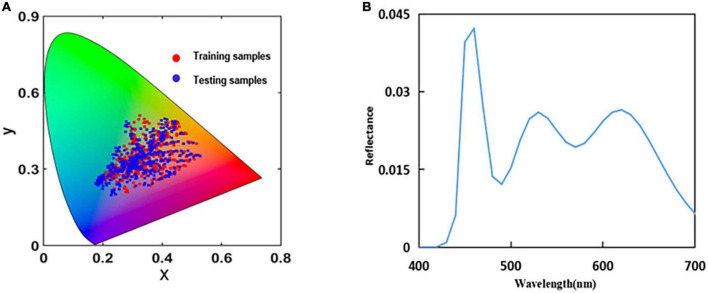
**(A)** Distribution of the sample points in the International Commission on Illumination (CIE)1964-XY chromaticity diagram and **(B)** real spectral power distribution of the light source.

Figure 6A shows the training and testing samples in the CIE1964-XY chromaticity diagram, where red and blue represent the training and testing samples, respectively. The power distribution of the light source in the shooting environment is measured using a CS2000 Spectroradiometer, as shown in Figure 6B.

**FIGURE 7:**
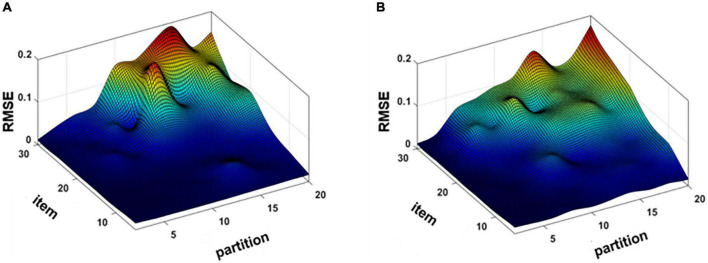
**(A)** Mean root-mean-square error (RMSE) map of the real experiment **(A)** K-mean angle similar (KS) and **(B)** K-means (KM).

Figure 7 shows the impact of the number of camera response extension items and partitions on the accuracy of recovery. The number of partitions was the same as in the simulation experiment, but the difference was the expansion items changed. The reason for this change might be the overfitting phenomenon. The optimal parameters of KS selected 2 partitions and the expansion items were 20, then the optimal parameters of KM selected 2 partitions, and the expansion items were 15 in a real experiment.

The experimental results of the real experiment are listed in Table 2. The results showed that the proposed method exhibited the best performance in terms of color difference, and the GFC and RMSE also presented the same results. Thus, the results showed that in the proposed method, the real experiment yielded better results, and it could be concluded that the proposed method in this study can be applied to a real scenario.

**TABLE 2:** Real spectral recovery results of the proposed and other existing methods.

	**CIE DE76**								
**Results**	**Li**	**Wang**	**Wu**	**Zhang**	**Cao**	**Kwon**	**Xu**	**KS**	**KM**

Mean	6.33	4.58	10.00	4.62	13.36	4.05	3.44	1.95	1.91
Maximum	33.08	13.76	25.75	14.65	41.89	18.39	19.35	10.16	8.87
Minimum	0.40	0.30	0.87	0.46	0.32	0.17	0.29	0.17	0.06

	**CIEDE2000**								
	**Li**	**Wang**	**Wu**	**Zhang**	**Cao**	**Kwon**	**Xu**	**KS**	**KM**

Mean	4.04	2.99	7.28	3.33	9.30	2.79	2.25	1.31	1.32
Maximum	11.65	10.99	21.65	7.37	28.44	9.29	11.55	8.04	3.92
Minimum	0.39	0.22	0.69	0.45	0.40	0.14	0.30	0.09	0.07

	**RMSE (%)**								
	**Li**	**Wang**	**Wu**	**Zhang**	**Cao**	**Kwon**	**Xu**	**KS**	**KM**

Mean	3.60	2.13	7.34	3.68	5.89	2.95	2.38	1.09	1.09
Maximum	11.85	7.38	22.08	15.97	23.89	13.81	15.65	5.44	4.73
Minimum	0.18	0.18	1.02	0.20	0.005	0.008	0.007	0.007	0.06

	**GFC (%)**								
	**Li**	**Wang**	**Wu**	**Zhang**	**Cao**	**Kwon**	**Xu**	**KS**	**KM**

Mean	99.44	99.81	99.70	99.85	98.23	99.88	99.91	99.95	99.96
Maximum	100.00	100.00	100.00	100.00	100.00	100.00	100.00	100.00	100.00
Minimum	45.09	97.69	96.62	98.02	86.85	97.65	98.64	97.98	99.09

**FIGURE 8:**
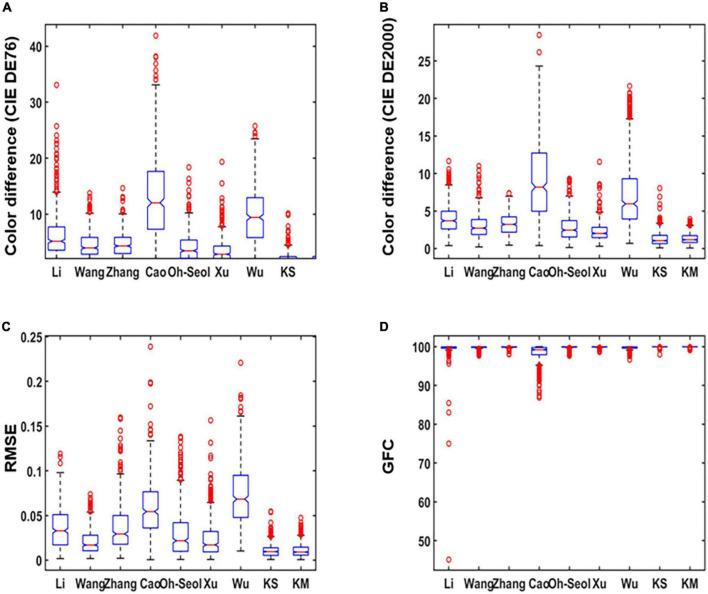
Boxplots of real experiment results: **(A)** The International Commission on Illumination (CIE) DE1976 color difference, **(B)** goodness of fit coefficient (GFC) CIE DE2000 color difference, **(C)** root-mean-square error (RMSE), and **(D)** GFC.

As can be seen from Figure 8, the smaller color difference in Figures 8A,B, smaller RMSE in Figure 8C, and larger GFC in Figure 8D show better performance of the proposed method. Thus, the boxplot distribution conclusion of our method presented better results than several other existing methods, which means recovery with less error and more application value.

**FIGURE 9:**
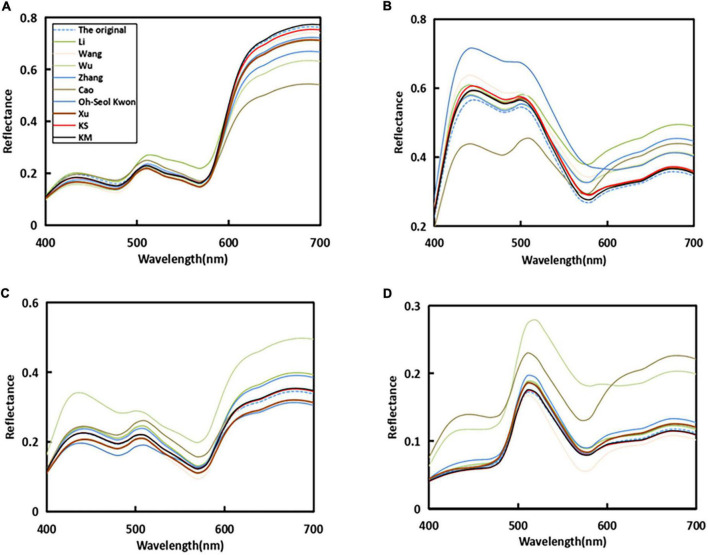
Reflectance recovery results from our proposed and existing methods with four randomly selected samples in a real experiment.

As can be seen from Figure 9, four random samples are selected in a real experiment. The similarity between the real and simulated experiments was that the recovery curves of the proposed method were more consistent with the original curves.k The higher similarity verified the excellence of the proposed method.

### Influence of color space

Since the way to acquire the response value was through the digital cameras, all weighting methods tended to use the more device-dependent RGB color space rather than other device-independent color spaces, such as CIE XYZ color space and CIE LAB color space. Three color spaces were used to explore the effect of color space on the proposed method. The RGB values of ANSI IT8.7/3 were acquired in a real experiment, which obeyed IEC about the sRGB standard. Then the RGB values were converted to CIE XYZ color space and CIE LAB color space (International Electrotechnical Commission [IEC], 2019), which were used to calculate the weighting function in three different color spaces, respectively.

**FIGURE 10:**
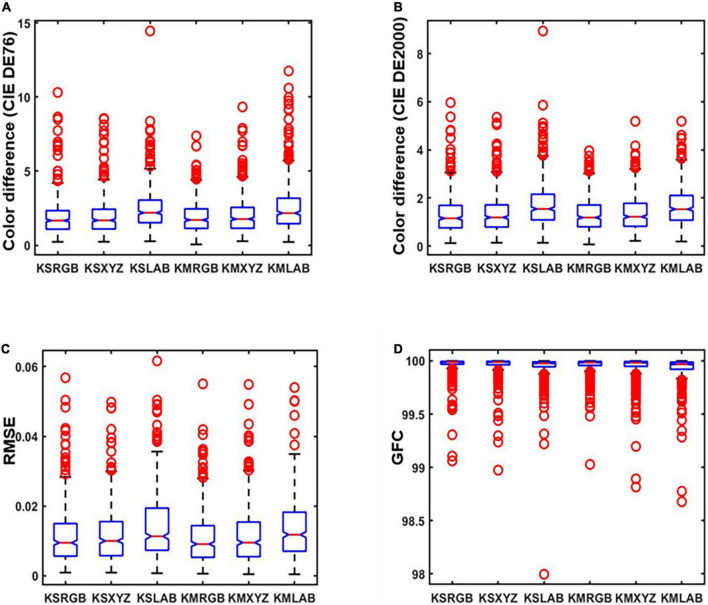
Boxplots of results in different color spaces: **(A)** The International Commission on Illumination (CIE) DE1976 color difference, **(B)** CIE DE2000 color difference, **(C)** root-mean-square error (RMSE), and **(D)** goodness of fit coefficient (GFC).

By the analysis of Tables 2, 3, the results showed that the accuracy of spectral recovery of the proposed method in three different color spaces is better than the other existing methods. To verify the spectral recovery accuracy further, Figure 10 presents the boxplots of results in three different color spaces. This study proposed an optimized method that verifies the effectiveness and robustness of spectral recovery accuracy under different color spaces.

**TABLE 3:** Spectral recovery results in different weighted color spaces.

	KM			KS		
	RGB	XYZ	LAB	RGB	XYZ	LAB
Mean ΔE	1.907	1.995	2.567	1.933	1.943	2.316
Max ΔE	8.871	9.329	11.753	7.281	8.5521	11.279
Mean GFC (%)	99.97	99.95	99.93	99.96	99.96	99.99
Max RMSE	0.055	0.055	0.054	0.047	0.042	0.050
Mean RMSE	0.011	0.011	0.013	0.011	0.012	0.012
DE2000 mean ΔE	1.311	1.365	1.658	1.315	1.36	1.525
DE2000 max ΔE	5.173	5.184	5.187	3.925	5.352	5.419

## Conclusion

This study has proposed an optimized method based on dynamic partitional clustering for spectral recovery from camera response values. The optimal local training samples were obtained using a dynamic and static combination clustering approach. After the proposed method was tested on both simulated and real experiments, the results showed that our proposed method outperformed other existing methods. The recovery accuracy of the proposed method using different color space weighting functions still outperformed the existing methods, which showed superior effectiveness and robustness. In future research, this method will also be applied to various fields, such as textiles, food, medicine, and cultural heritage.

## Data availability statement

The original contributions presented in this study are included in the article/supplementary material, further inquiries can be directed to the corresponding author/s.

## Author contributions

All authors listed have made a substantial, direct, and intellectual contribution to the work, and approved it for publication.
